# Non-linear models for the detection of impaired cerebral blood flow autoregulation

**DOI:** 10.1371/journal.pone.0191825

**Published:** 2018-01-30

**Authors:** Max Chacón, José Luis Jara, Rodrigo Miranda, Emmanuel Katsogridakis, Ronney B. Panerai

**Affiliations:** 1 Departamento de Ingeniería Informática, Universidad de Santiago de Chile, Santiago, Chile; 2 Department of Cardiovascular Science, University of Leicester, Leicester, United Kingdom; 3 Biomedical Research Centre, University of Leicester, Glenfield Hospital, Leicester, United Kingdom; Ehime University Graduate School of Medicine, JAPAN

## Abstract

The ability to discriminate between normal and impaired dynamic cerebral autoregulation (CA), based on measurements of spontaneous fluctuations in arterial blood pressure (BP) and cerebral blood flow (CBF), has considerable clinical relevance. We studied 45 normal subjects at rest and under hypercapnia induced by breathing a mixture of carbon dioxide and air. Non-linear models with BP as input and CBF velocity (CBFV) as output, were implemented with support vector machines (SVM) using separate recordings for learning and validation. Dynamic SVM implementations used either moving average or autoregressive structures. The efficiency of dynamic CA was estimated from the model’s derived CBFV response to a step change in BP as an autoregulation index for both linear and non-linear models. Non-linear models with recurrences (autoregressive) showed the best results, with CA indexes of 5.9 ± 1.5 in normocapnia, and 2.5 ± 1.2 for hypercapnia with an area under the receiver-operator curve of 0.955. The high performance achieved by non-linear SVM models to detect deterioration of dynamic CA should encourage further assessment of its applicability to clinical conditions where CA might be impaired.

## Introduction

New advances in the continuous, non-invasive measurement of arterial blood pressure (BP) and cerebral blood flow (CBF), have facilitated the evaluation of dynamic cerebral autoregulation (CA) at the bed side or during physiological maneuvers [1]. CA is the mechanism responsible for maintaining CBF relatively constant, despite changes in mean BP in the range 60–150 mmHg [2–3].

Dynamic CA has been defined as the transient response of CBF, usually estimated as CBF velocity (CBFV) with transcranial Doppler, to a sudden change in BP. Although initially proposed as the CBFV response to a BP drop induced by the rapid release of compressed thigh cuffs [4], a number of other maneuvers have been proposed to induce changes in BP to provoke corresponding changes in CBFV [5–8]. Of considerable interest is the demonstration that dynamic CA can be assessed using spontaneous fluctuations of BP and CBFV, by assuming that these signals correspond to the input and output variables, respectively, of a linear or non-linear model whose properties can describe the physiological or pathological characteristics of the CBF regulatory mechanisms. Although the use of spontaneous fluctuations has clear advantages, such as maintaining physiological stability and facilitating clinical studies in critically ill patients, its reliability has been questioned due to the limited signal-to-noise ratio (SNR) resulting from relatively small fluctuations of BP and CBFV.

To address the SNR limitation, without resorting to special maneuvers to increase BP variability, investigators have proposed more advanced models that could overcome the limitations of standard linear transfer function analysis (TFA), which is the classic analytical approach used in this context [9]. These models have included dedicated neural networks, Wiener-Laguerre kernels, multivariate modeling, principal dynamic modes, multi-modal pressure-flow or wavelet analysis [10–16]. We have previously reported that support vector machines (SVM) [17] have particular advantages to model dynamic CA, including its potential to capture non-linear behavior, one aspect of dynamic CA that has not received enough attention. More recently, we have also reported on the possibility of quantifying dynamic CA performance using a ‘model-free’ index of autoregulation (mfARI) [18], which could be a more robust metric than the more established dynamic autoregulation index (ARI) proposed by Tiecks et al. [4]. In the present study, we have compared mfARI values estimated from non-linear SVM models with both the gain and phase estimates provided by TFA and the classic ARI index of Tiecks et al. to assess the relative diagnostic performance of these methods. For this purpose, we compared baseline recordings of BP and CBFV from healthy subjects with similar recordings from the same subjects in hypercapnia, which is often used as a surrogate of impaired CA [1, 19–20]. The relevance of this investigation is to allow a controlled assessment of the diagnostic performance of different methods for the quantification of dynamic CA using the receiver-operating characteristic curve (ROC) approach before translation to clinical applications.

In summary, we tested the hypothesis that using mfARI to measure the quality of responses derived from SVM non-linear models provides greater discrimination of reduced dynamic CA in comparison to the combination of TFA and the standard ARI.

## Materials and methods

### Subjects and measurements

This study uses healthy subject data from previous studies with measurements obtained at rest, during baseline physiological conditions and hypercapnia induced with 5% carbon dioxide (CO_2_) breathing [19, 21]. In summary, subjects aged 18 years or older, free from hypertension, diabetes, migraine, epilepsy, or any other cardiovascular or neurological disease, were studied in a temperature controlled laboratory free from distraction. Subjects were asked to refrain from ingesting alcohol or caffeinated products in the 12 h preceding their participation. Ethical approval was obtained from the Southampton and South West Hampshire Research Ethics Committee A (10/H0502/1), and written informed consent was obtained in all cases.

Measurements were made in the supine position. CBFV was recorded in the right middle cerebral artery with transcranial Doppler (Companion III, Viasys Healthcare, San Diego, CA, USA) using a 2 MHz transducer. BP was measured non-invasively using arterial volume clamping of the digital artery (Finometer, Finapres Medical System, Amsterdam, The Netherlands). Delivery of 5% CO_2_ in air was achieved with a face mask (Vital Sing, Totowa, NJ, USA), which was connected to a CO_2_ delivery subunit. The subunit comprises a Y valve that controls whether CO_2_ or air is being administrated and a 200 I Douglas bag used to store the CO_2_/air mixture. The face mask was also connected to a capnograph (Datex Normocap 200, Helsinki, Finland) to measure end-tidal CO_2_ (EtCO_2_) continuously. A 3-lead surface electrocardiogram (ECG) was also recorded.

Baseline values of CBFV, BP and EtCO_2_ were recorded for an initial period of 5 min. with subjects breathing normal air, after all variables were stable for at least 15 min. This was followed by a 5 min. recording with each subject breathing the CO_2_/air mixture.

Signals were sampled at a rate of 200 Hz. All signals were filtered in both directions using an eight-order Butterworth low-pass filter with a cut off frequency of 20 Hz. ECG was used for detecting the beginning and end of each cardiac cycle and mean beat-to-beat values were estimated for BP, CBFV, HR and EtCO_2_. These physiological parameters were interpolated using a third order polynomial and resampled to 0.2 s to create a uniform time base. Finally, the signals were sub-sampled to 2 Hz and its amplitude normalized within the [0–1] interval for use by the SVM.

### Transfer function analysis

The Welch’s periodogram method was used to estimate auto-power and cross-spectral power densities in order to calculate a transfer function (TF) using BP as input and CBFV as output. Each 5 min. recording was broken down in 102.4 s (512 samples) segments of data and FFT transformed with 50% overlap, following a multiplication by a Hanning window as recommended by the Cerebral Autoregulation Research Network [9]. The resulting estimates of gain and phase were averaged for the low frequency (LF: [0.07–0.2] Hz) and very low frequency (VLF: [0.02–0.07] Hz) ranges, for comparison with the ARI estimates as described below.

### Classic dynamic autoregulation index

The method proposed by Tiecks et al. [4] assesses changes in CBFV in response to changes introduced in BP by the sudden release of inflated bilateral thigh cuffs. The relationship between the BP and CBFV signals is described by a second order differential equation with three parameters, which was used to generate responses for specific trios of parameter values as ten grading templates associated to index values between 0 (absence of autoregulation) and 9 (best autoregulation). The ARI value of an actual CBFV response to a thigh-cuff maneuver is determined by selecting the template that best fits the observed signal. We used continuous ARI values obtained by interpolation as indicated in Chacon et al. [22].

### Model-free autoregulation index

**Fig 1:**
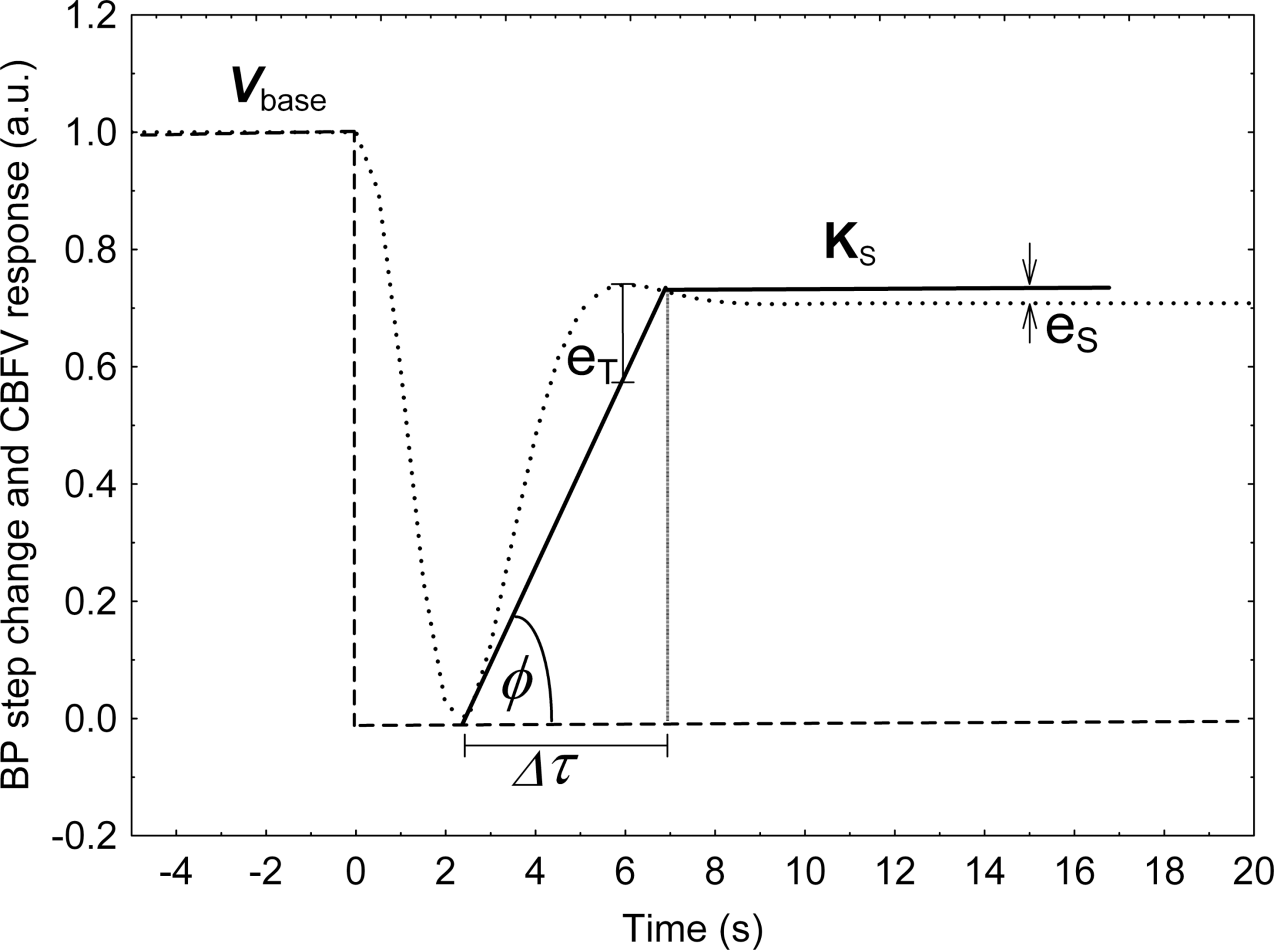
mfARI parameters. Characterization of a typical model's CBFV response (dotted line) to a BP negative step (dashed line). Δ*τ* is the duration of the transient response. *V*_base_ is the baseline level, which is used to normalize the signal before determining the slope of the straight line that characterize its transient response (thick solid line). The horizontal thick solid straight line is the representation of the constant steady state response (*K*_s_). The angle between the line of transient CBFV response and line of BP recovery is the parameter *ϕ*. *e*_T_ and *e*_S_ are examples of the errors in the transient and steady state phase respectively, their sum is minimized to fit the best set of straight lines.

Unlike the classic ARI, this index does not need to be calculated by fitting templates produced by an arbitrary mathematical model and it is not limited to responses from a linear system. Thus, mfARI values can be used to characterize responses from both linear and non-linear models. This index was designed to be calculated from the response to a thigh-cuff maneuver, but it can also be used to evaluate step responses generated by a model, as exemplified in Fig 1. The method runs an optimization process to fit the best pair of straight lines to the CBFV response by minimizing the sum of *e*_T_ and *e*_S_ errors (Fig 1). One straight line characterizes the transient state (usually with a positive slope) and the other, horizontal line, represents the steady-state response of the system (a constant, *K*_s_). The rise time (Δ*τ*) of the former straight line, i.e. the duration of the transient state, corresponds to the first parameter estimated from the step response; the second parameter is the constant *K*_s_ itself. Finally, a third parameter is determined that corresponds to the angle (*ϕ*) between the straight line that represents the transient state of CBFV and the straight line that represents the transient BP signal. To obtain index values in the same scale of [0–9] used by the classic ARI, we used templates, as proposed by Tiecks et al. [4], to gauge the three mfARI parameters (Δ*τ*, *K*_s_ and *ϕ*) from each one and generated a regression equation to relate them to the corresponding ARI values. In this way, for each model's response to a BP step it is possible to obtain an mfARI value by using this regression equation. The advantages of mfARI, in terms of correctness and reproducibility, as well as its theoretical foundations, have been reported previously [18].

### Proposed models

Support Vector Machines have been used to obtain non-linear models of the ABP-CBFV dynamic relationship [17]. The fundamentals of SVM are presented in S1 Appendix. SVM are static models that relate an input vector to an output real value. In order to capture the temporal relationship of two or more signals, it is necessary to add external delays or recurrences. Specifically, we used *ν* Support Vector Regression (*ν*-SVR) to develop dynamic univariate models with BP as input and CBFV as output of two types: Non-linear Finite Impulse Response (NFIR) models and Non-linear Autoregressive (NAR) models. The structures of these types of models are shown in Eqs (1) and (2) respectively, in which *p*(*t*) is the BP value at time instant *t*, $\hat{v}(t)$ is the predicted CBFV at time instant *t*, and F represents a non-linear function.

$$\hat{v}(t)=F\left(p(t),p(p-1),\cdots ,p(t-n_{p})\right)$$ (1)

$$\hat{v}(t)=F\left(\hat{v}(t-1),\hat{v}(t-2),\cdots ,\hat{v}(t-n_{v}),p(t),p(p-1),\cdots ,p(t-n_{p})\right)$$ (2)

Each pair of signals, i.e. simultaneous recordings of spontaneous fluctuations of BP and CBFV of one subject, was separated into two sections: the training segment, consisting of the first two and half minutes of the signals, and the validation segment, with the last two and half minutes. All models were trained with the former section using the one-step-ahead prediction strategy, and validated with the latter fragment following the model predictive output strategy, in which the model predicts the complete CBFV validation segment for the unseen validation BP segment. The search for both the delays in the BP signal and the recurrences was made empirically. The hyper-parameters of non-linear *ν*-SVR were bounded by grid search. Then the process was repeated after interchanging the training and validation segments.

The efficiency of a model was determined with the Pearson’s correlation coefficient (CC) between the real CBFV signal and the CBFV estimated by the model. The best model for each subject is the one with the highest CC in the validation segment. But high correlation is not enough to guarantee that a model’s response has physiological plausibility. To solve this problem, we implemented a computational routine, based on the indications suggested by Ramos et al. [23], that automatically discards models that generate non-physiological responses. Training and validating subroutines were implemented using the *R environment* [24] to *libsvm* [25] in package *e1071* [26].

### Statistical analysis

Data normality was tested using Shapiro-Wilk’s statistic. Paired comparisons were made with the Student's t-test or the Wilcoxon test as appropriate.

We evaluated each approach separately by analyzing three different linear mixed-effect models (LMEM) that included, following current recommendations [27–28], both random intercepts and random slopes by subject. LMEM were built using the *lmerTest* R package [29] and estimated marginal means, standard errors, confidence intervals and contrast p-values (employing the Kenward−Roger approximation for degrees of freedom) were obtained with the *lsmeans* [30] package. The LMEM to analyze the goodness-of-fit of the models used as fixed effects the model structure (NFIR or NAR) and the subject’s condition (normal or hypercapnia), as well as their interaction. In this case, correlation coefficients were previously converted to standard z scores for arithmetic manipulation using Fisher's transformation. The LMEM to compare autoregulation index values also considered model structure and subject’s condition, but the index type (classic ARI or mfARI) was also introduced as a fixed effect. The LMEM for TFA included the TF coefficient (gain or phase), the frequency range (VLF or LF) and the subject’s condition. In this case, individual TF coefficient values had to satisfy recommended statistical criteria imposed on the coherence function to be included in the study [9].

The discriminatory ability of the different approaches was compared in terms of the area under the ROC curve (AUC). ROC curves were obtained from autoregulation index values by using numbers in [0–9] as discrimination thresholds, while we used the subject’s mean values of TF gain and phase for this. AUC values achieved by each method were compared with the non-parametric test provided in the *pROC* R package [31], which is based on the bootstrap method (5,000 iterations). In all cases, *p* < 0.05 was considered statistically significant.

## Results

Good quality recordings were obtained for 45 subjects aged 31 ± 12 years, in both baseline and hypercapnia. Grand means and standard deviations (SD) of systemic and cerebrovascular parameters, including indices of dynamic CA across subjects, are given in Table 1. The Shapiro-Wilks test indicated lack of normality in the case of BP. In the TFA analysis, one baseline case and two cases of hypercapnia did not meet the bounds imposed by the coherence function and were removed from the study. Noteworthy, most parameters in Table 1 showed highly significant changes as a result of hypercapnia. Subject-specific TF coefficients are detailed in S1 File.

**Table 1:** Grand mean ± SD of physiological variables, TF coefficients and predicted dynamic CA index values in baseline and hypercapnia.

Variable	Baseline	Hypercapnia	*p*-value
BP (mmHg)	94.5 ± 18.4	99.4 ± 19.4	.012
EtCO_2_ (mmHg)	40.6 ± 3.1	45.8 ± 2.9	< .001
CBFV (cm s^-1^)	57.4 ± 2.9	65.8 ± 14.8	< .001
TF gain_VLF_ (cm s^-1^ mmHg^-1^)	1.1 ± 0.6	1.0 ± 0.4	.287
TF gain_LF_ (cm s^-1^ mmHg^-1^)	1.6 ± 0.7	1.2 ± 0.4	< .001
TF Phase_VLF_ (rad)	0.7 ± 0.2	0.5 ± 0.2	< .001
TF Phase_LF_ (rad)	0.4 ± .4	0.4 ± 0.3	.462
NAR (mfARI)	5.9 ± 1.5	2.5 ± 1.2	< .001
NAR (ARI)	4.7 ± 1.7	2.1 ± 1.2	< .001
NFIR (mfARI)	5.7 ± 1.8	2.4 ± 1.8	< .001
NFIR (ARI)	4.7 ± 2.0	1.3 ± 1.2	< .001

BP: Arterial blood pressure, EtCO_2_: End-tidal carbon dioxide, CBFV: Cerebral blood flow velocity, LF: Low frequency (range), VLF: Very low frequency (range), NFIR: Non-linear Finite Impulse Response (model), NAR: Non-linear autoregressive (model), ARI: (dynamic) Autoregulation index, mfARI: Model-free ARI. *p*-values from paired Student’s t-test or Wilcoxon signed-rank test for difference between baseline and hypercapnia.

**Fig 2:**
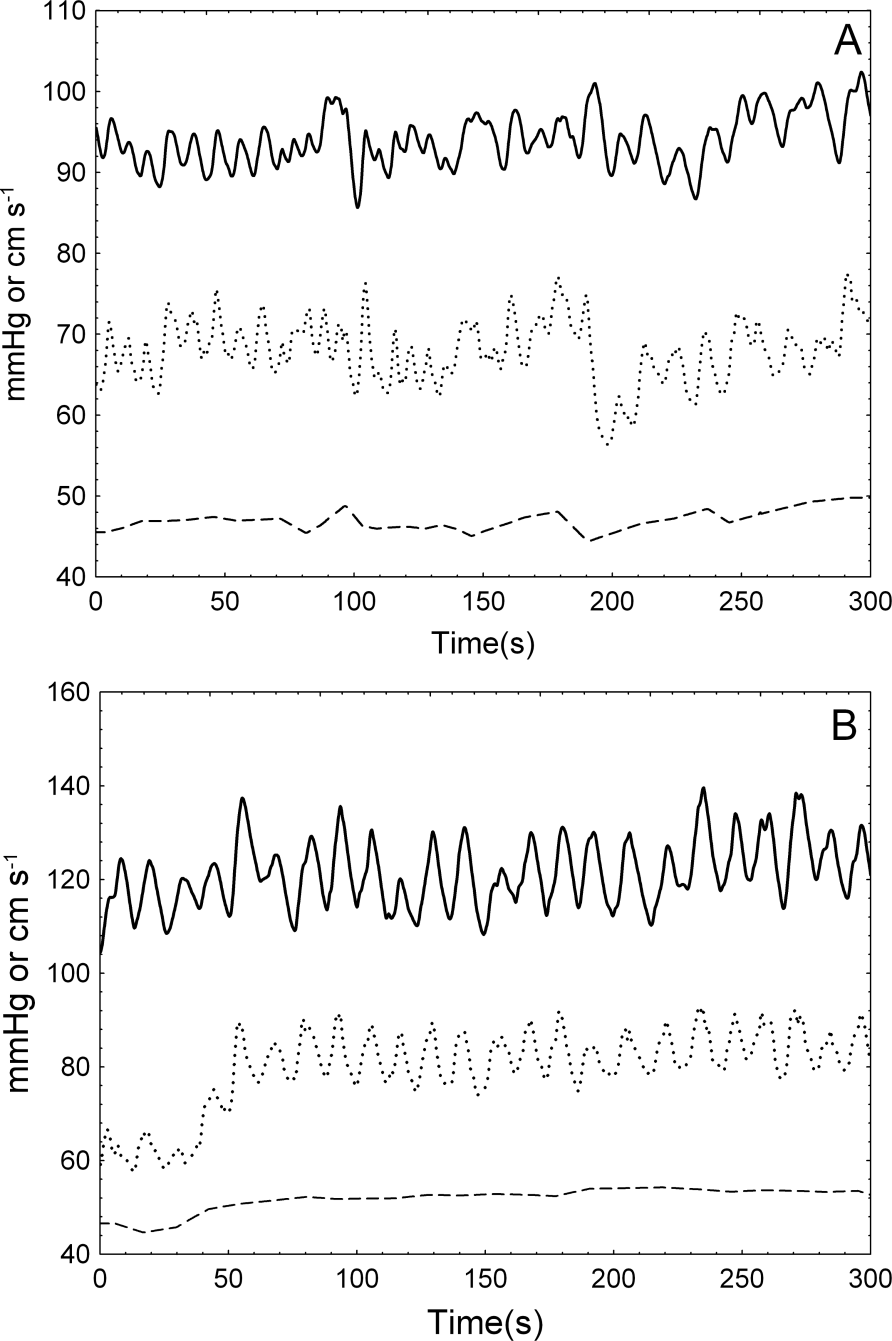
Signals in spontaneous fluctuations. Recordings of spontaneous BP (solid line, mmHg), CBFV (dotted line, cm/s), and EtCO_2_ (dashed line, mmHg) from a female 29-years-old volunteer, in baseline (A) and hypercapnia (B). An increase in all signals can be observed in the latter after 50 seconds.

Spontaneous fluctuations of BP, EtCO_2_ and CBFV for a representative subject are shown in Fig 2 where the characteristic slow rise in CBFV is observed following breathing 5% CO_2_ in air (Fig 2B).

**Fig 3:**
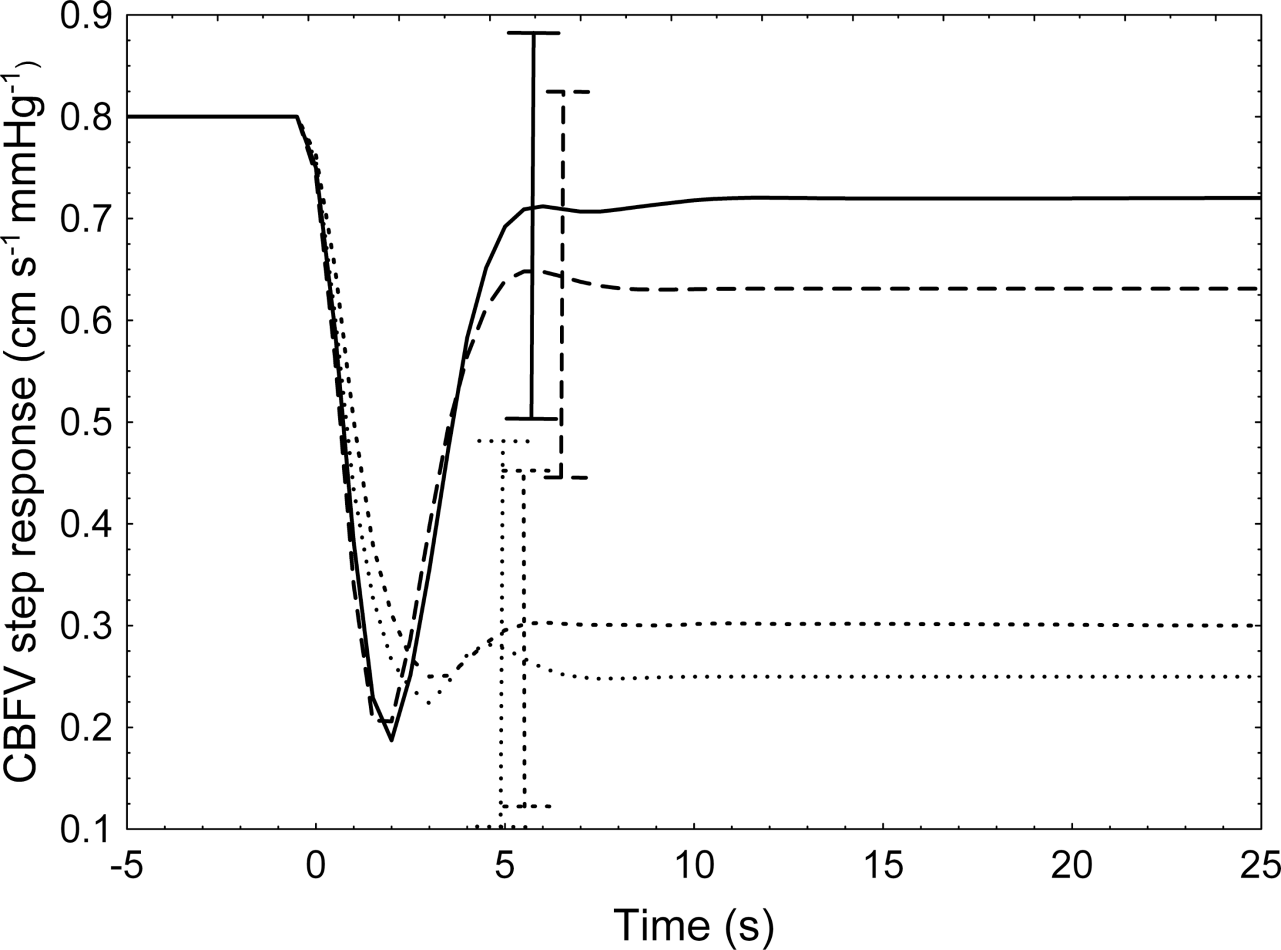
Mean step responses. Each curve shows the averaged step responses generated for each subject by NAR models (solid line) and NFIR models (long dashed line) in baseline, and NAR models (dashed line) and NFIR models (dotted line) in hypercapnia. Error bars represent ± SD.

**Fig 4:**
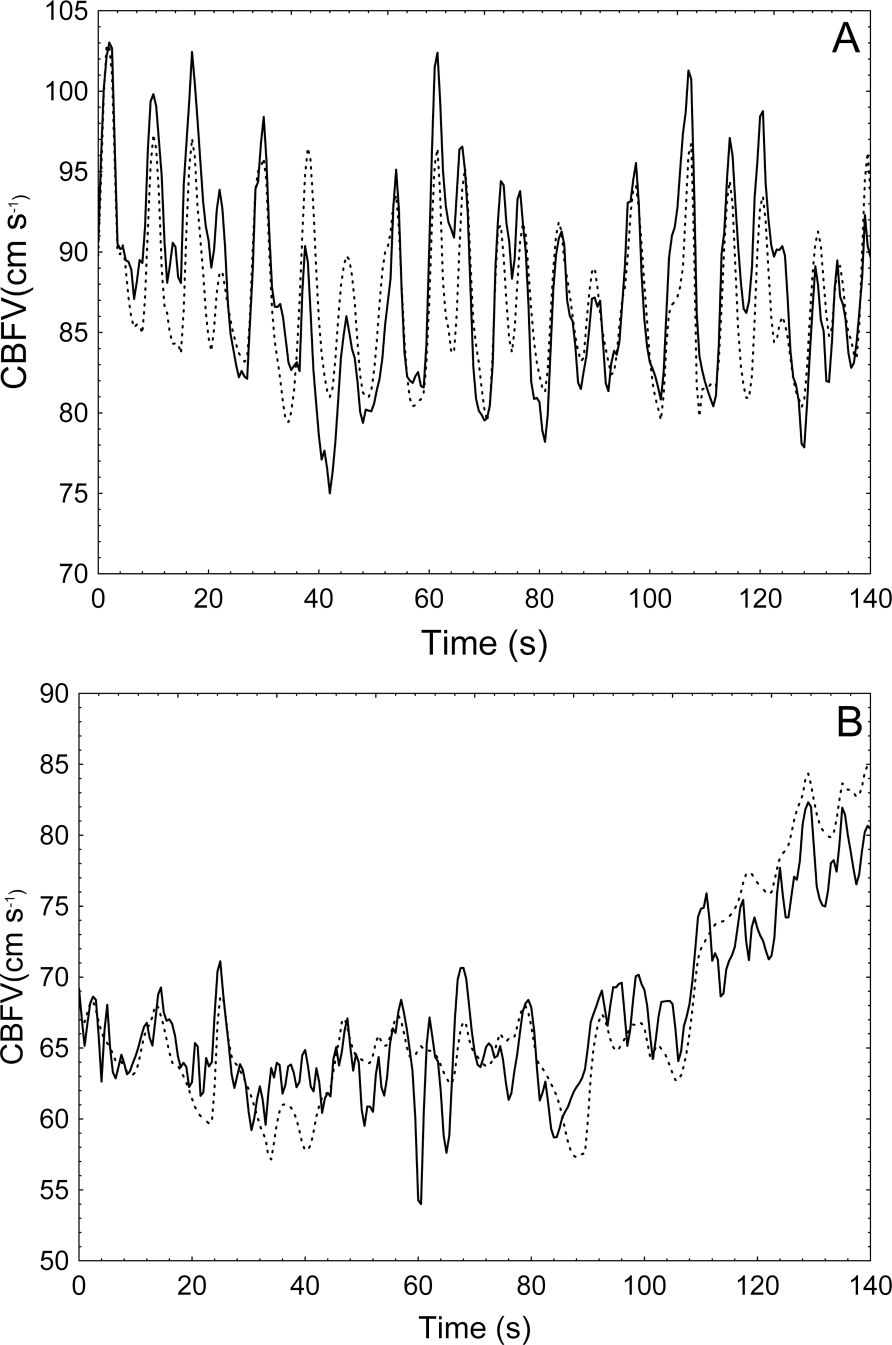
Comparison of the actual and predicted CBFV signals. The CBFV signal predicted by a non-linear NAR model (dotted line) compared with the actual CBFV signal (solid line) of a 26-years-old male volunteer, in baseline (A) and hypercapnia (B).

All NAR models exhibited acceptable responses to the input step of BP, while responses with plausible physiological patterns could not be obtained with NFIR models for two subjects in hypercapnia. Global mean step responses, for each type of model and condition, are given in Fig 3, in which the difference in temporal pattern due to hypercapnia can be clearly appreciated. In all cases, significant test correlation coefficients were obtained. Individual responses can be found in S2–S5 Files. Fig 4 illustrates a comparison of the actual CBFV with the predicted response by a NAR model for a representative subject. NFIR models reached similar levels of correlation in both conditions (*p* = .198), which were significantly lower than the correlations obtained by NAR models (*p* < .003). The best goodness-of-fit was achieved by NAR models in hypercapnia (*p* < .001). A summary of the *ν*-SVR hyper-parameters used by the models selected for each subject can be found in Table 2. Detailed information is provided in S6–S9 Files. In addition, these models presented lower variance, reflected in lower coefficients of variation for mfARI, ranging from 25.4% (NAR, baseline) to 75% (NFIR, hypercapnia).

**Table 2:** *ν*-SVR hyper-parameters and validation correlations coefficients exhibited by the models selected for both non-linear structures in both conditions.

Model	*n*_*p*_	*n*_*v*_	*C*	*υ*	*γ*	CC
NAR baseline	5 [1–8]	2 [1–6]	4602.7 ± 6419.0	0.48 ± 0.30	0.17 ± 0.34	0.74–0.80
NAR hypercapnia	3 [1–8]	1 [1–6]	4636.5 ± 6195.1	0.46 ± 0.28	0.40 ± 0.60	0.84–0.89*
NFIR baseline	8 [1–8]	-	4604.6 ± 6644.5	0.44 ± 0.30	0.39 ± 1.27	0.67–0.75
NFIR hypercapnia	1 [1–8]	-	4521.1 ± 6363.2	0.46 ± 0.33	1.19 ± 4.87	0.61–0.72

*n*_*p*_: Mode [range] of the number of external delays of the BP signal used as model’s input, *n*_*v*_: Mode [range] of the number of external recurrences (CBFV) used as model’s input, *C*: Mean ± SD of the values used as penalties of the error term, *υ*: Mean ± SD of the values used as lower bounds of the fraction of support vectors, *υ*: Mean ± SD of the values used as lower bounds of the fraction of support vectors, *γ*: Mean ± SD of the values used as the radial base kernel scope, CC: Estimated confidence interval for mean correlated coefficient, NFIR: Non-linear Finite Impulse Response (model), NAR: Non-linear autoregressive (model).

* Significantly higher (p < .001) compared to the other models.

**Fig 5:**
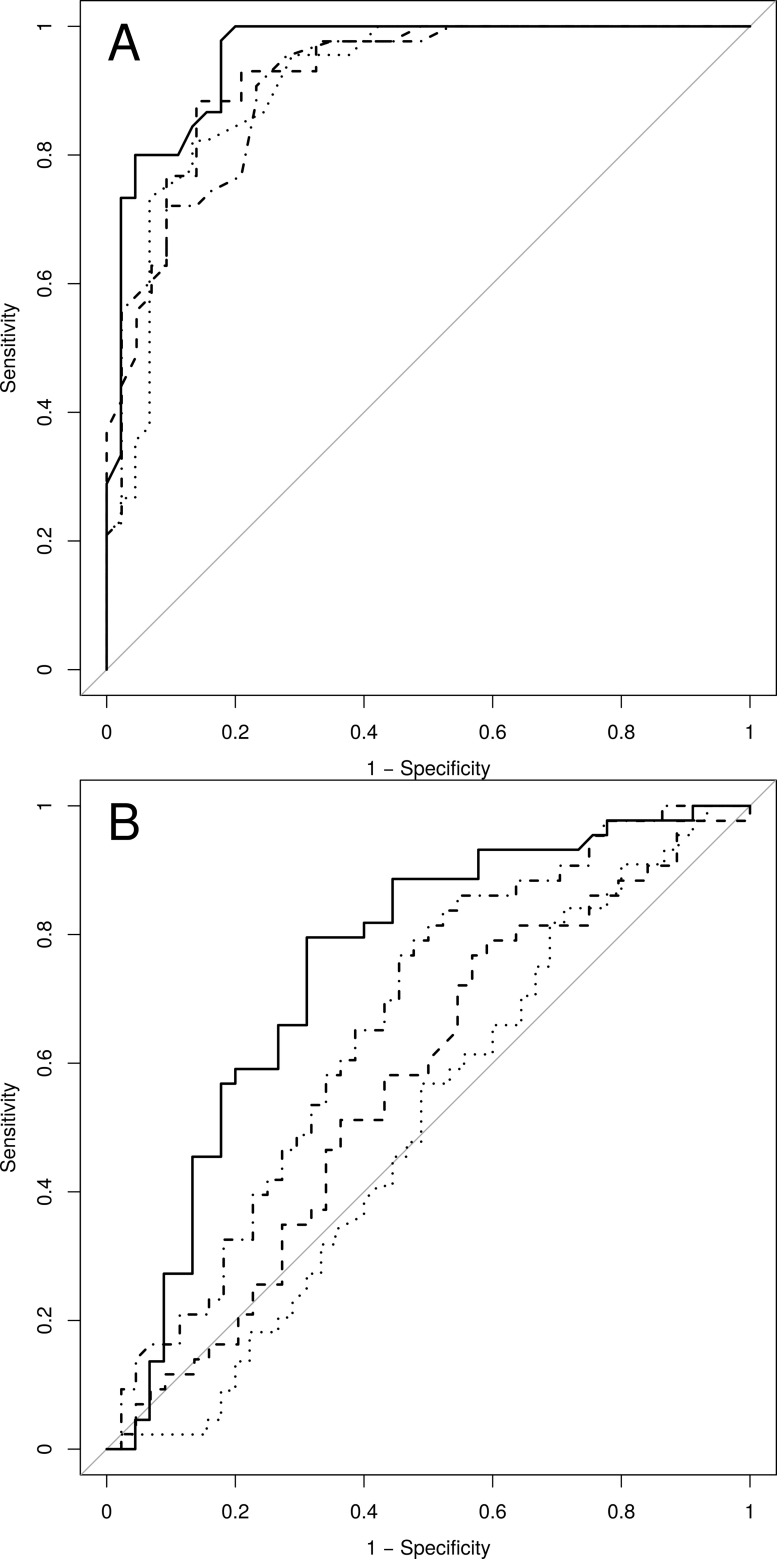
ROC curves. In subplot (A) ROC curves achieved by non-linear models with classic ARI values for NFIR (dotted line) and NAR models (dashed line), and with mfARI values for NFIR (long dashed) and NAR models (solid line). ROC curves for TF are presented in subplot (B) with TF gain_VLF_ (dotted line), TF phase_LF_ (dashed line), TF gain_LF_ (dot dashed line) and TF phase_VLF_ (solid line).

ROC curves produced by the non-linear models (Fig 5A) and by TFA (Fig 5B) are clearly distinct. AUC values for each approach are summarized in Table 3. mfARI was significantly better than ARI (*p* = .022) to detect changes in dynamic CA induced by hypercapnia with NAR models, but not with NFIR model (*p* = .431). Neither autoregulation index (*p* = .178 for mfARI, *p* = .748 for ARI) showed significant differences in AUC between NAR and NFIR structures. For TFA, the coefficient with the largest AUC was phase_VLF_, followed by gain_LF_ and phase_LF_ (Table 3). Nonetheless, phase_VLF_ AUC value still resulted significantly lower than for both types of non-linear models using mfARI (*p* = .007 with NFIR, *p* < .001 with NAR). Notably, Gain_VLF_ was very close to the line of indifference (AUC = 0.506).

**Table 3:** AUC values obtained by each approach when discriminating between baseline and hypercapnia.

Model/TF coef.	mfARI	ARI	LF	VLF	*p*-value
NAR	0.955*	0.914			.022
NFIR	0.925*	0.909			.431
TF gain			0.667	0.506	< .001
TF phase			0.568	0.749	.023
*p*-value	.178	.748	.321	.001	

TF: Transfer function, ARI: (dynamic) Autoregulation index, mfARI: Model-free ARI, LF: Low frequency (range), VLF: Very low frequency (range), NAR: Non-linear autoregressive (model), NFIR: Non-linear Finite Impulse Response (model).

* Significantly higher than best AUC achieved by TF coefficients.

## Discussion

### Main findings

Hypercapnia has often been adopted as a safe and convenient maneuver to reduce the efficacy of CA, which could be regarded as a surrogate of CA impairment as observed in many clinical conditions [1, 10, 17, 19, 32–33]. A number of studies have adopted the ROC as a ‘gold standard’ for assessing the classification effectiveness of dynamic CA parameters between these two conditions [19, 34]. Compared to values of ROC AUC previously reported, the proposed methods showed considerable improvements in the ability to detect changes in dynamic CA efficiency due to hypercapnia, reaching AUC values over 0.9.

*ν*-SVR has been widely used in many different fields, but more often in the form of ‘static’ models. To allow the representation of dynamic behavior, as is the case of dynamic CA, we had to add either a moving average or a recursive structure, as described in Eqs (1) and (2). Of considerable relevance is the demonstration that, although higher values of AUC were obtained with the autoregressive form (NAR), it was not significantly better to corresponding mfARI values derived with the moving average structure (NFIR). Since NAR are computationally much more demanding due to the need to optimize an additional parameter (*n*_*v*_ in Table 2), the NFIR alternative represents an easier and faster approach to non-linear modeling using the SVM formalism. On the other hand, the superiority of the new mfARI, in comparison with the classical ARI [4], was only manifested when extracted from NAR models. This is an intriguing finding that deserves further investigation. Moreover, given the limited reproducibility of the ARI reported in previous studies, the improved intra-session reproducibility of mfARI warrants further investigation in inter-session reproducibility studies [7, 24].

### Non-linear models of dynamic CA

CBFV variability using EtCO_2_ as a co-variate has already been studied, especially relevant are the works of Mitsis et al. [10–11] and Chacón et al. [17], in which the influence of both BP and EtCO_2_ on CBFV was analyzed using non-linear models. However, none of these studies attempted the evaluation of the discriminatory ability of these models to detect changes in dynamic CA efficiency during hypercapnia. Neither did they compare the non-linear models with linear methods such as TFA. Chacón et al. also used models based on the SVM formalism and demonstrated that the NAR structure was able to capture a complex interaction between the two input variables, namely BP and EtCO_2_, on the generation of the output CBFV signal, something that linear models cannot achieve. Specifically, the quality of the autoregulatory responses from the NAR models decreased as the levels of EtCO_2_ increased (see figure 6 in [17]), in concordance with previous direct observations on subjects. In contrast, non-linear models in this study are univariate and, thus, only captured the relationship between the BP signal and the CBFV signal. Nonetheless, they were able to detect alterations in the CBFV dynamics and produce the expected patterns for autoregulatory responses after introducing changes in EtCO_2_, as depicted in Fig 3.

The best linear discriminator was mean TF phase_VLF_ with an AUC of 0.749, which coincided with the value of 0.746 obtained using classic ARI values estimated from the step responses of transfer functions for subjects in the same conditions [19, 32] (of which we shared 29 cases). This attribute of the TF phase was already described by Birch et al. [35], who reported a significant reduction of this coefficient during hypercapnia in contrast to baseline values.

In respect to discriminatory ability, the great superiority of using non-linear models could be demonstrated, as the two structures tested, in combination with mfARI, were significantly better than any TF coefficient.

### Study limitations

Although we have consistently used the term ‘hypercapnia’, capnography is just an indirect, noninvasive measurement of the levels of arterial CO_2_. Nonetheless, its reliability for measurements performed supine at rest has been well established and it has become an essential part of patient care worldwide [36].

Despite inspiration of 5% CO_2_ in air being a well-established method to induce a deterioration in CA, the participants were healthy subjects, and it would be of great interest to evaluate the discriminatory ability of these methods with patients suffering from diseases known to impair dynamic CA. For this purpose, it would also be relevant to broaden the age range of the control group.

The classification of subjects in baseline and hypercapnia was much superior to all previous studies that can currently be found in the literature. Regardless of the great discriminatory ability offered by non-linear models, especially in combination with the model-free dynamic autoregulation index, it continues to be a ‘black-box’ method, which contributes little information to the understanding of the underlying mechanisms of dynamic CA.

The comparison with an established linear method showed that non-linear modeling played a key role in achieving the high discriminatory ability of the proposed method. However, these ‘non-linear’ characteristics refer to the appropriate fitting of the model to the data, and not necessarily to the characteristics of dynamic CA. For example, the non-linearity adopted by the models with radial base functions could be a non-linear compensation to numerically fit the data better from a more non-stationary than non-linear phenomenon. Thus, with the type of SVM used in this study it is not possible to definitively establish the characteristics that differentiate these models from the linear methods. Further studies, aiming at discovering what valuable information can be obtained from these black-box methods, would be necessary to shed light on these questions.

In a similar direction, to become a diagnostic tool with clinical applications, the proposed method uses endpoint signals that have been demonstrated to be feasible to obtain in a clinical setting, namely BP and CBFV. Unfortunately, state-of-the art technology does not yet allow accessible and noninvasive measurements that could shed light on the underlying phenomena implicated in deterioration of dynamic CA and further research in this direction is therefore an important priority.

## Conclusion

Non-linear models of the BP-CBFV relationship provided superior ability to detect changes in dynamic cerebral autoregulation induced by hypercapnia, compared to the most commonly used linear approach represented by transfer function analysis. Best results were obtained with a new index of dynamic CA, that is ‘model free’ (mfARI) and hence does not depend on *a priori* conditions of the temporal pattern of the CBFV step response. The methodology used to evaluate the approach clearly showed its clinical potential, demonstrating its ability to detect diminished dynamic CA responses, even when the cause of the alteration is not being directly observed. Further work is needed to validate this approach in a larger number of subjects, involving a wider range of ages and phenotypes, as well as to extend the reported findings to alterations of cerebral hemodynamics introduced by other factors or pathological conditions.

## Supporting information

S1 Appendix Support vector machines. Theoretical fundamentals of Support Vector Machines and Regression.
(DOCX)

S1 File Transfer function coefficients. Gain and phase, in the low and very low frequency ranges, for each subject, in baseline and hypercapnia.
(CSV)

S2 File NFIR step responses in baseline. Step responses produced by the Non-linear Finite Impulse Response models selected for each subject in baseline.
(CSV)

S3 File NFIR step responses in hypercapnia. Step responses produced by the Non-linear Finite Impulse Response models selected for each subject in hypercapnia.
(CSV)

S4 File NAR step responses in baseline. Step responses produced by the Non-linear Autoregressive models selected for each subject in baseline.
(CSV)

S5 File NAR step responses in hypercapnia. Step responses produced by the Non-linear Autoregressive models selected for each subject in hypercapnia.
(CSV)

S6 File NFIR parameters in baseline. *ν*-SVR hyper-parameters of the Non-linear Finite Impulse Response models selected for each subject in baseline.
(CSV)

S7 File NFIR parameters in hypercapnia. *ν*-SVR hyper-parameters of the Non-linear Finite Impulse Response models selected for each subject in hypercapnia.
(CSV)

S8 File NAR parameters in baseline. *ν*-SVR hyper-parameters of the Non-linear Autoregressive models selected for each subject in baseline.
(CSV)

S9 File NAR parameters in hypercapnia. *ν*-SVR hyper-parameters of the Non-linear Autoregressive models selected for each subject in hypercapnia.
(CSV)
