# Supplementary material for: Non-linear models for the detection of impaired cerebral blood flow autoregulation
Source: PLoS One. 2018 Jan 30;13(1):e0191825. doi: 10.1371/journal.pone.0191825 (PMC5790248; doi:10.1371/journal.pone.0191825)
Supplement: S1 Appendix — Theoretical fundamentals of Support Vector Machines and Regression. (DOCX) [file pone.0191825.s001.docx]

**S1 Appendix – Support Vector Machines**

In this work we use SVM for regression (SVR), specifically the *ν*-SVR algorithm [37] introduced by Schölkopf et al. The algorithm is based on the results of the statistical learning theory presented by Vapnik [38], which uses regression as the fitting of a tube of radius *ε* to the data. Vapnik's *ε*-SVR estimates the $\vec{w}$ values to get the function

*f* (*x*) = ($\vec{w}$· $\vec{x}$) + *b,* $\vec{w}$, $\vec{x}$ $\in$ **R**^n^, b $\in$ **R**. (A.1)

The algorithm is implemented by minimizing to which a penalization is added for leaving points outside the tube (identified by slack variables *ξ*). In this way the empirical risk to be minimized is given by equation (A.2), where the *C* is a constant that determines a compromise between the model's complexity and the points that remain outside the tube,

minimize $\theta(\vec{w},\xi)=\frac{1}{2}+C\left(l\varepsilon\nu+\sum_{i=1}^{l} \xi_{i}\right)$ (A.2)

where *l* represents the total number of cases (dataset's dimension). The variation introduced by Schölkopf et al. [37] consisted in using a single parameter *ν*, and making the radius of the tube *ε* dependent of *ν*, as shown in equation (A.2), so that only this parameter controls the total proportion of support vectors that are used in the minimization calculations. We use *ν* values in {0.1, 0.2, …, 0.9} for our experiments.

The solution of this optimization problem is found in the dual space when the Kuhn-Tucker conditions are applied. To solve a non-linear regression problem it is sufficient to replace the dot product between two original independent variables  by a kernel function. This function performs the dot product in a space of higher dimensionality, so that it assures the linearity of the regression function in the new space, via a non-linear transformation. Several functions can be used as kernels. In our case we used the most common non-linear function with a single parameter, namely the Gaussian kernel or radial base function, which only requires the adjustment of the *γ* parameter to determine the specific non-linearity required (equation A.3).

(A.**3**)

Therefore, it is necessary to estimate three parameters from the data to obtain a trained *ν*-SVR non-linear model, namely *C*, *ν* and *γ*.

**References**

[37] Schölkopf B, Smola AJ, Williamson RC, Bartlett PL. New Support Vector Algorithms. Neural Comput. 2000;12:1207–45.

[38] Vapnik VN. The Nature of Statistical Learning Theory. 1995; Springer.
